# Concomitant Gluteal and Thigh Compartment Syndrome Following Atraumatic Injury

**DOI:** 10.7759/cureus.17009

**Published:** 2021-08-08

**Authors:** Molly McNamee, Matthew Wolfers, Mhamad Faour, Lisandro Montorfano, Stephen J Bordes

**Affiliations:** 1 Anatomical Sciences, St. George's University School of Medicine, St. George's, GRD; 2 Surgery, Cleveland Clinic Florida, Weston, USA; 3 Surgery, Louisiana State University Health Sciences Center, New Orleans, USA

**Keywords:** compartment syndrome, alcohol abuse, drug abuse, gluteal compartment, thigh compartment, substance abuse

## Abstract

Gluteal compartment syndrome is a rare diagnosis associated with pelvic trauma and subsequent surgical intervention. Herein, we discuss the case and management of gluteal and thigh compartment syndrome following prolonged immobilization secondary to alcohol. To our knowledge, we present the first case of concomitant gluteal and thigh compartment syndrome following atraumatic injury.

## Introduction

Lower extremity compartment syndrome is a well-known and common entity following traumatic and atraumatic events. Gluteal compartment syndrome is a rare diagnosis typically associated with pelvic trauma (i.e., fractures and crush injuries) but can be seen as a complication following improper surgical positioning and prolonged immobilization secondary to alcohol intoxication. While gluteal compartment syndrome has been reported in the literature, simultaneous gluteal and thigh compartment syndrome has not. We discuss the presentation and management of gluteal and thigh compartment syndrome following prolonged immobilization secondary to alcohol intoxication.

## Case presentation

A 32-year-old man with a past medical history of alcohol abuse was brought to the emergency department (ED) by emergency medical services after being found unconscious in his front yard. The patient admitted to drinking heavily and using intravenous drugs the night prior. He fell asleep in a chair for an unknown amount of time and was found lying on his right side with a wallet in his back pocket.

On initial presentation, the patient was hemodynamically stable and alert with a Glasgow Coma Scale (GCS) of 15. His main complaint was low back pain and a rash on the right hip. Physical exam showed erythema and tenderness over the right buttock and lateral aspect of the right thigh. He had a full range of motion without deformity. Lower extremity pulses were 2+ bilaterally with 3 out of 5 strength in hip flexion, knee extension, plantar flexion, and dorsiflexion of the right lower extremity (RLE). The remainder of the physical exam was unremarkable.

Initial workup was significant for a blood potassium level of 7.9 and electrocardiogram (ECG) showing sinus tachycardia with nonspecific T-wave abnormalities. Additionally, a serum creatine kinase >10,000 U/L, lactic acid of 3.7 mmol/L, and a creatinine of 3.08 mg/dL were suggestive of acute renal failure secondary to rhabdomyolysis. A Foley catheter was inserted into the bladder with a return of 30 mL of pink-tinged urine. The patient was administered 60 mg of kayexalate, 2 g of calcium gluconate, albuterol, 50% dextrose hypertonic fluid (D50), and 10 units of insulin to treat hyperkalemia. The patient was initially admitted to the medical intensive care unit (ICU) for resuscitation and management of metabolic derangement.

In the ICU, the patient complained of RLE weakness and numbness along the right flank that extended distally to the right toes with pain out of proportion. However, pulses remained palpable (2+) in the RLE. Compartment pressures were evaluated using a Wick catheter, which measured 50 mmHg in the right buttock and 48 mmHg in the lateral right thigh compartment. Computed tomography (CT) of the RLE showed subcutaneous edema in the lateral soft tissues. Acute compartment syndrome was confirmed, and the patient was taken to the operating room for emergent decompressive fasciotomy of the right buttocks and thigh.

**Figure 1:**
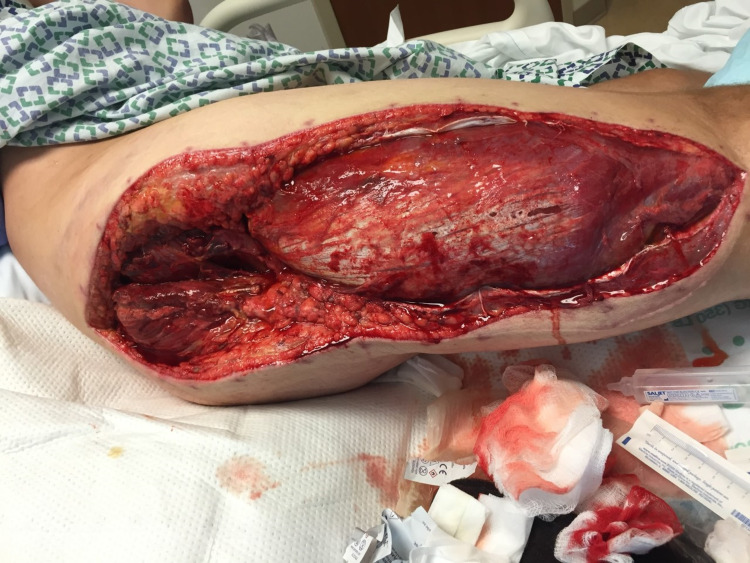
Anterior thigh compartment fasciotomy.

An incision was made from the posterior superior iliac spine down to the greater trochanter of the femur and extended to the lateral femoral condyle. The anterior thigh compartment was extremely tense upon exposure and was successfully decompressed with the incision to the femoral condyle. The vastus lateralis muscle was swept anteriorly to access the posterior compartment fascia (Figure 1). Intraoperative pressure measurement of the medial compartment was 6 mmHg, so the medial compartment was spared. There was no evidence of myonecrosis upon visual inspection of the compartments. The incision was irrigated and approximated with staples and vessel loops for a shoelace partial closure. Xeroform dressing was applied and covered with gauze and an ACE wrap bandage.

**Figure 2:**
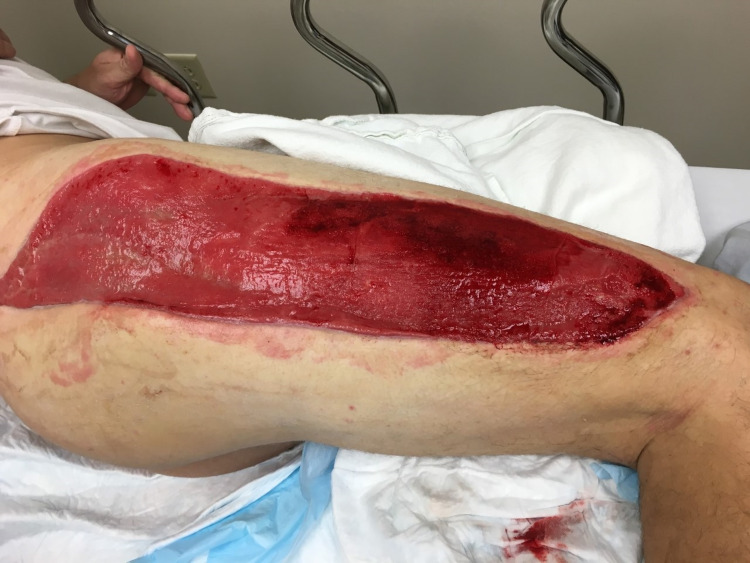
Anterior fasciotomy undergoing negative pressure therapy.

**Figure 3:**
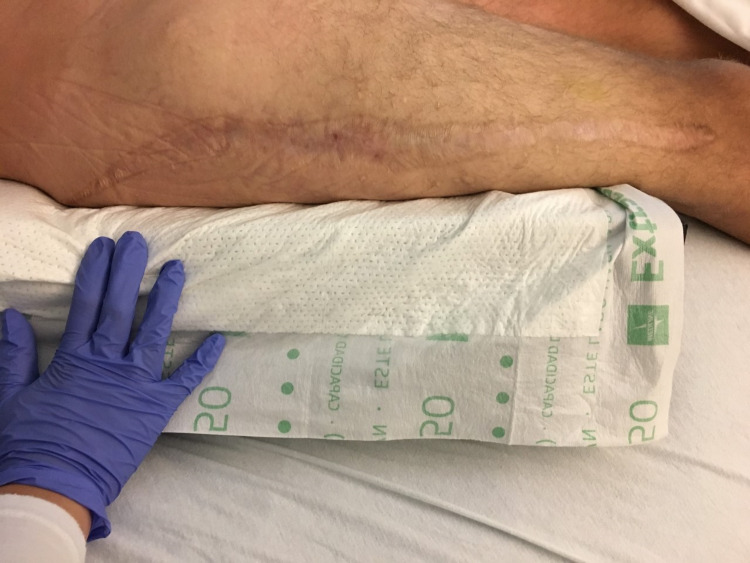
Well-healed fasciotomy two years postoperatively.

On postoperative day 3, the patient returned to the operating room for a second exploration. The right gluteus and thigh compartments were inspected for signs of ischemia. Muscles were soft and well-perfused. The incision was irrigated, and a negative pressure wound vacuum (-75 mmHg) was applied over the fasciotomy site. The postoperative course was uneventful, and the patient’s acute kidney injury resolved without complication. The patient was ultimately discharged on postoperative day 14 with referrals for home physical therapy and wound care. After discharge, the patient followed up closely for wound inspection and negative pressure dressing changes (Figure 2). Six weeks after surgery, the patient underwent formal wound closure at an outside hospital. He was seen two years postoperatively with a well-healed incision and fully functioning extremity (Figure 3).

## Discussion

Acute compartment syndrome is considered a surgical emergency that can be life and limb threatening. The gluteal region is a rare anatomic location for compartment syndrome. However, the majority of the reported cases of gluteal compartment syndrome were a direct result of prolonged immobilization [1-5]. The gluteal region has been documented to form three separate compartments in cadaveric studies. The compartments are separated by the gluteus maximus, gluteus medius, and minimus, and the tensor fascia latae, which is continuous with the thigh. The thigh compartments are divided into the anterior (femoral nerve, sartorius muscle, and quadriceps muscles), middle (obturator nerve, external obturator, gracilis, adductor longus, adductor brevis, and adductor minimus), and posterior (sciatic nerve, adductor magnus, biceps femoris, semitendinosus, and semimembranosus) compartments. Due to the separation of the fascial components of the gluteal area and thigh, it is rare to see a simultaneous compartment syndrome in both locations. In this case, we report a rare simultaneous gluteal and thigh compartment syndrome in a patient with prolonged immobilization of the lower extremity following drug and alcohol abuse. The patient recovered without any complications due to prompt intervention.

The most commonly reported causes of prolonged immobilization leading to gluteal compartment syndrome in the literature include heavy substance use and surgical positioning [2-7]. Traumatic injuries, typically resulting from a crush injury to the lower lumbar spine, pelvis, and buttocks area, comprise approximately 20% of cases. Prolonged extrication time is further recognized as a known association in these situations.

Physical examination, including intramuscular compartment pressure measurements, remains the best tool for accurate diagnosis. In this case, compartment pressure measurements confirmed the clinical suspicion following the insidious progression and evolution of symptoms in our patient. Patients present with severe pain in the gluteal regions and lower extremity paresthesia. A tense and painful buttock, ecchymosis, and/or Morel-Lavallee lesions may be noted on physical exam. Passive motion of the hip exacerbates the pain. Intra-compartmental pressure measurements can be a useful adjunct, especially in cases of limited exam and with an unconscious or uncooperative patient [8].

Prompt decompressive fasciotomy is the standard of care for compartment syndrome. Delayed diagnosis can lead to complications such as sciatic nerve palsy and kidney failure among others [4,5,9]. Postoperative adjunct management options have been reported to promote wound healing such as negative pressure wound vacuum and hyperbaric oxygen, though limited data are available to support the latter. Hayden et al. discussed the use of hyperbaric oxygen for the management of gluteal compartment syndrome postoperatively to reduce edema, salvage marginal ischemic tissue, and accelerate granulation of the wound bed, allowing earlier wound closure and/or grafting [10].

## Conclusions

Gluteal and thigh compartment syndrome is a rare entity associated with prolonged immobilization due to intoxication and surgical positioning. To our knowledge, this case is the first of its kind in the literature. Compartment syndrome can be a life- and limb-threatening condition and is thus a surgical emergency. Prompt surgical intervention with decompressive fasciotomy remains the mainstay in the management of this condition. Providers should remain vigilant as the key to an uncomplicated recovery is early recognition and decompression.

## References

[1] Iizuka S, Miura N, Fukushima T, Seki T, Sugimoto K, Inokuchi S (2011). Gluteal compartment syndrome due to prolonged immobilization after alcohol intoxication: a case report. Tokai J Exp Clin Med.

[2] Mitsiokapa EA, Mavrogenis AF, Salacha A, Tzanos G (2013). Acute lumbosacral plexopathy from gluteal compartment syndrome after drug abuse: a case report. J Surg Orthop Adv.

[3] Khalifa R, Craft MR, Wey AJ, Thabet AM, Abdelgawad A (2020). Missed positional gluteal compartment syndrome in an obese patient after foot surgery: a case report. Patient Saf Surg.

[4] Liu HL, Wong DSY (2009). Gluteal compartment syndrome after prolonged immobilisation. Asian J Surg.

[5] Adrish M, Duncalf R, Diaz-Fuentes G, Venkatram S (2014). Opioid overdose with gluteal compartment syndrome and acute peripheral neuropathy. Am J Case Rep.

[6] Rocos B, Ward A (2017). Gluteal compartment syndrome with sciatic nerve palsy caused by traumatic rupture of the inferior gluteal artery: a successful surgical treatment. BMJ Case Rep.

[7] Chew MH, Xu GG, Ho PW, Lee CW (2009). Gluteal compartment syndrome following abdominal aortic aneurysm repair: a case report. Ann Vasc Surg.

[8] Dilernia FD, Zaidenberg EE, Gamsie S, Zamboni DET, Carabelli GS, Barla JD, Sancineto CF (2016). Gluteal compartment syndrome secondary to pelvic trauma. Case Rep Orthop.

[9] Engelund D, Kjersgaard AG (1991). [Acute compartment syndrome]. (Article in Danish). Ugeskr Laeger.

[10] Hayden G, Leung M, Leong J (2006). Gluteal compartment syndrome. ANZ J Surg.

